# Elimination of Bloodstream Infections Associated with *Candida albicans* Biofilm in Intravascular Catheters

**DOI:** 10.3390/pathogens4030457

**Published:** 2015-06-29

**Authors:** Freshta Akbari, Birthe Veno Kjellerup

**Affiliations:** 1 Department of Biological Sciences, Goucher College, Baltimore, MD 21204, USA; E-Mail: frakb001@mail.goucher.edu; 2 Department of Civil and Environmental Engineering, University of Maryland at College Park, College Park, MD 20742, USA

**Keywords:** antibiotic lock technique, biofilm, bloodstream infections, *Candida albicans*, chelating agents, intravascular catheters

## Abstract

Intravascular catheters are among the most commonly inserted medical devices and they are known to cause a large number of catheter related bloodstream infections (BSIs). Biofilms are associated with many chronic infections due to the aggregation of microorganisms. One of these organisms is the fungus *Candida albicans*. It has shown to be one of the leading causes of catheter-related BSIs. The presence of biofilm on intravascular catheters provide increased tolerance against antimicrobial treatments, thus alternative treatment strategies are sought. Traditionally, many strategies, such as application of combined antimicrobials, addition of antifungals, and removal of catheters, have been practiced, but they were not successful in eradicating BSIs. Since these fungal infections can result in significant morbidity, mortality, and increased healthcare cost, other promising preventive strategies, including antimicrobial lock therapy, chelating agents, alcohol, and biofilm disruptors, have been applied. In this review, current success and failure of these new approaches, and a comparison with the previous strategies are discussed in order to understand which preventative treatment is the most effective in controlling the catheter-related BSIs.

## 1. Introduction

Microorganisms mainly exist in biofilms in natural environments, including the external environment (lakes, sediments, *etc.*) and locations in the human body. Biofilms are defined as communities of microorganisms that are encapsulated in a self-produced extracellular polymeric substance (EPS) attached to a surface [1,2] (Organisms prefer the biofilm mode compared to the planktonic mode, as they are able to exchange nutrients and genetic materials, as well as provide protection to one another [2]. It has become particularly evident to the medical community that biofilms are the causative agents of various nosocomial and chronic infections, many of which are resistant to current antimicrobial therapies. One of the many nosocomial infections developed in a hospital is catheter-related bloodstream infections (BSIs) [3]. In U.S., approximately 500,000 central-line associated BSIs are reported every year. However, the estimated number of infections is likely higher than reported [4]. Due to longer hospital stays and more extensive treatments, the estimated cost of catheter-related BSIs is approximately $33,000–$65,000 per case [4]. Collectively, these infections lead to high morbidity, mortality, and costs for health care delivery system.

**Figure 1:**
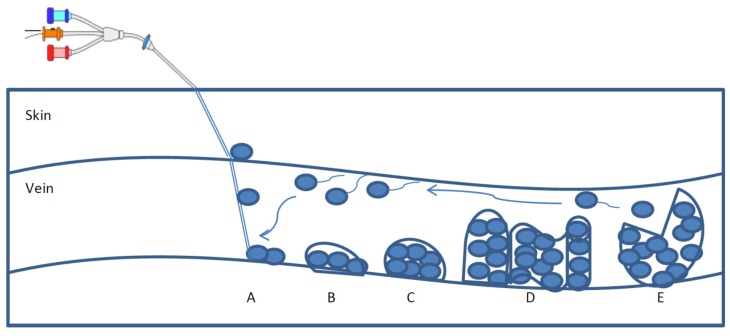
The life cycle of biofilms as complex matrices that provide a protective environment for pathogens. The five stages are A: contamination and initial attachment, B: permanent attachment, C: colonization and primary maturation, D: secondary maturation and biofilm development, and E: dispersion of the planktonic bacteria and re-attachment leading to new sites of biofilm formation and risk of infection.

There are a number of microbial species causing nosocomial bloodstream infections. The most common of these are *Staphylococcal* and *Candida* species that are related to catheter-related BSIs [5]. Specifically, *Candida* species are reported to be responsible for 5% to 71% of mortality and morbidity in BSIs, respectively [6]. Among the *Candida* species, *C. albicans* has shown to be commonly associated with BSIs, where it forms biofilms on the surface of intravascular catheters [2]. *C. albicans* can cause candidiasis displaying mucosal and systemic infections [7]. An infection develops when microorganisms from a patient’s skin at the insertion site of the catheter attach to the surface of the indwelling catheter to form a biofilm [1] (Figure 1). Following the biofilm formation, the microbial cells from the biofilm can disperse into the bloodstream leading to serious infections [1]. Detachment of aggregates of these cells, the production of endotoxins, or other pyrogenic substances lead to the symptoms of disease in patients [3].

Although *C. albicans* is among the top four leading causes of catheter related BSIs, other *Candida* species, such as *C. parapsilosis*, *C. Pseudotropicalis*, and *C. glabrata*, are capable of forming biofilm. However, they are less pathogenic compared to *C. albicans* [8]. *Candida* species are characterized as commensal organisms, but they can become pathogenic at times, where the host immune defense is not fully active [9]. As an opportunistic pathogen, *C. albicans* easily adapts to its surrounding environment with the help of its recognition proteins (adhesions), morphogenesis (conversion from yeast to hyphal form), and its proteolytic and lipolytic enzymes [5]. They primarily infect patients who are in an immune-compromised state, have diabetes mellitus, inserted medical device, and/or intravenous drug fluid feeding their body [9]. Similar to most microorganisms, *C. albicans* exist in biofilm form that not only provides a protected environment, but it can also allow for horizontal gene transfer that potentially code for antibiotic resistance. This can result in antibiotic concentrations up to 1000-fold greater than needed for treatment of their planktonic counterparts [3,8,10]. Thus, *C. albicans* have a higher resistance to antifungal and antimicrobial approaches making it difficult to prevent them from causing BSIs. Therefore, to improve patient outcome and to reduce healthcare costs, there is considerable interest in lowering the incidence of these infections and seeking potential solutions. Although this is challenging, various prevention strategies have demonstrated success that requires further research. The objective of this paper is to evaluate present prevention strategies, their limitations, and to introduce new technologies.

## 2. Biofilm Formation and Characteristics

Development of catheter-related BSIs is associated with the biofilm formation on the device. *C. albicans* biofilms are complex microbial communities that possess unique characteristics, which need to be considered when presenting biofilm prevention solutions. *C. albicans* biofilm forms in three different stages starting when (1) the organisms attach to the surface of the catheter; (2) they subsequently secret extracellular polymers; and (3) they form a 3-D structure that surrounds and protect the organisms [9,11]. Contamination of the catheter surface at the time of insertion can introduce microorganisms into the catheter lumen leading to infections [8]. As the biofilms mature, various morphologies and components such as polysaccharides and proteins are observed [9]. Carbohydrates are one of the main components of *C. albicans* biofilm and Chandra *et al.* (2001) [12], confirmed in a study that addition of saliva and glucose enhanced the biofilm formation on a denture acrylic model [12].

Andes *et al.* (2004), described *C. albicans* biofilm in a rat model as a bilayer structure, which was verified both by fluorescent and scanning electron microscopy thus explaining the increased pathogenicity caused by this organism [8,9,10]. Further, the inner portion of the biofilm was thin, whereas the outer portion was dense and contained both the yeast and hyphal form of *C. albicans* [10]. In other studies, confocal laser scanning electron microscopy (CLSM) revealed *C. albicans* biofilms as a heterogeneous 3-D structure that contained water channels similar to most other biofilms [8,11]. While the basic characteristics of *C. albicans* biofilm in laboratory settings are similar to the biofilms that other species produce, evaluation of infected tissues exhibited multiple morphological forms of *C. albicans* including but not limited to hyphae or pseudohyphae and oval budding yeast [8]. These morphological aspects of the biofilm contribute to the stability of the fungal communities. Douglas (2003) [8] reported when a hyphae-negative mutant was grown, it contained a basal yeast layer, while the yeast-negative mutant created hyphal-like biofilms similar to the wild-type ones [8]. Later, it was observed that the yeast-negative mutants were less structurally stable indicating that the basal yeast layer assisted in anchoring and supporting the biofilm onto the surface [8]. These biofilm characteristics are crucial in proposing solutions to prevent infections caused by *C. albicans*.

## 3. Current Preventative Approaches and Their Effectiveness

Traditionally, various antimicrobials and antifungals have been extensively used for prevention of bloodstream catheter infections. However, *C. albicans* biofilm structure and up-regulation of the resistance gene expressions allow them to be resistant to antimicrobial regiments as high as 30 to 2000 times that of planktonic cells [5,8,9,11]. Since *C. albicans* is a commensal fungal organism, the inhibition of its fungal activity could lead to decreased infections in the bloodstream [2]. Antifungal drugs have also been broadly used for the purpose of finding solutions to catheter related BSIs. 

The biofilm structure contributes to the exhibited tolerance of *C. albicans* towards a wide spectrum of antimicrobial and antifungal agents. It has been suggested that the matrix of the biofilm acts as a barrier to prevent penetration of the agents and thus limit their effect on the microbial communities [8]. To test this, biofilm communities that were grown in shaking conditions were observed to have disrupted matrices that decreased their susceptibility (20%) to amphotericin B as opposed to those grown under static conditions [13]. As mentioned earlier, the presence of a thick EPS layer, as the biofilm matures, contributes to the resistance level against antifungals as was seen by Kuhn *et al.* (2002) [14], when fluconazole, nystatin, cholorohexidine, terbenafine, and amphotericin B were tested [14]. Additionally, expression of genes that code for a multidrug efflux pump could give rise to a multidrug resistant phenotype [8]. The CDR and MD genes encode for the two efflux pumps of the ATP-binding cassette (ABC) and other facilitators respectively [8]. These two efflux pumps were shown to be activated during biofilm formation and a mutation in one or both of these genes resulted in increased susceptibility to fluconazole by *C. albicans* in its planktonic form [15]. Thus, there are multiple factors that influence whether *C. albicans* show resistance to antimicrobial and antifungal agents.

### Antimicrobials

In 1996, Raad *et al.* [1] reported that catheters that were coated with antimicrobials of minocycline and rifampin inhibited the activity of *C. albicans* *in vitro* compared to the catheters coated with chlorohexidine gluconate and silver sulfadiazine [1]. The inhibitory activity was evaluated using the Kirby-Bauer method, in which the zone of inhibition of each catheter treated with a combination of anti-infective agent was measured in mm. They ascribed the higher efficacy of minocycline and rifampin to their synergistic effect that increased their inhibition activity against *C. albicans* related infections. Additionally, minocycline-rifampin possesses a broad spectrum activity against *Candida* species and a number of bacterial species, which could be useful in treating polymicrobial biofilms [1]. This combination also showed to have a longer half-life of antimicrobial activity (25 days) as opposed to three days for catheters that contained chlorohexidine gluconate and silver sulfadiazine [1]. Even though the minocycline-rifampin combination acted as inhibitory agents against *C. albicans* biofilm, a comparative evaluation of their activity against another strategy such as chelating agent was not done. Therefore, an accurate conclusion of antimicrobial effectiveness cannot be drawn. In another study, Hanna *et al.* (2006) [16], demonstrated that central venous catheters coated with gendine were more effective in preventing biofilm formation of *C. albicans* than those treated with antibiotics, or platinum, silver and carbon [16]. Gendine, an antiseptic that contains gentian violet and chlorhexidine, was more effective against *Candida* species. It was further mentioned that antimicrobial therapies of minocycline-rifampin have limitations, when gram-negative or *Candida* species were present in catheters [16].

More recently Maki (2010) [4] has observed that placing antimicrobial luer-activated connectors coated with nanoparticle silver into the surface of catheters killed a significant amount of *C. albicans*, as well as five other examined bacterial species [4]. According to Maki (2010) [4], application of nanoparticle silver into the catheters reduced the presence of *C. albican*s by 99.9% [4]. Knowing that high silver concentrations can negatively affect the health of individuals, this strategy should be applied with caution. Though, Maki (2010) [4], mentioned that the bactericidal ionic silver particles released from the surface of catheters was approximately 0.05 ng/mL, which is greatly below the mean blood level of 0.2–5 ng/mL in an untreated healthy individual [4]. Thus the silver particles in the fluid path were thought to add to the inhibitory activity of antimicrobial agents to prevent biofilm formation. Although antimicrobial agents have been used to treat intravascular infections, the challenge is that they only exhibit short term suppression and they lead to possible drug-resistance that needs to be addressed [16].

In addition to the various antimicrobials, a number of synthetic biofilm dispersing agents can be applied for dispersion of biofilms. Among them are synthetic 2-Aminoimidazole (2-AI) that naturally can be found in marine products [17]. Examples of 2-AI compounds are ageleferin and oroidin that have shown to disperse biofilms from of a number of Gram-positive and Gram-negative pathogenic bacteria such as methicillin-resistant *Staphylococcus aureus* (MRSA), *Acinetobacter baumannii*, and *Pseudomonas aeruginosa* [17,18,19]. Su *et al.* (2011) [20] observed that a library of 4, 5-di-substititued-2-aminoimidazole-triazole conjugates (2-AIT) inhibited biofilm formation by MRSA and *A. baumannii* that are opportunistic pathogens [20]. These bacteria can enter the body via open wounds or catheters. In a study conducted by Thompson *et al.* (2012) [19], a 2-AI compound inhibited the response regulator protein BfmR in *A. baumannii,* which controls its biofilm formation [19]. As 2-AIs inhibit biofilm formation, there is a possibility to utilize them as anti-biofilm agents for treatment after further understanding of their systems and therapeutic advantages in different strains of pathogenic bacteria has been obtained.

Synthetic antimicrobial peptides can also be applied as alternatives to antimicrobials for treatment of biofilms. Among them, some of the most studied peptides that have been effective against *C. albicans*’ biofilms are reported to be LL-37, human β-defensin-3 (hBD-3), KABT-AMP, and KSL-W [21,22,23,24]. Antimicrobial peptides (AMPs) are cations that inhibit formation of cell-walls, proteins, and nucleic acids [21]. For example, LL-37 has shown to have antimicrobial activity against bacteria and fungi such as *C. albicans* [21]. The adhesion step in infection is necessary as the carbohydrate and protein-rich cell-wall of *C. albicans* needs to interact with the host epithelial cells. If this cell-cell contact is inhibited, biofilm formation and infectivity of *C. albicans* will be inhibited as well [21]. Tsai *et al.* (2011) [21] further reported that low concentrations of LL-37 interrupt the adhesion of *C. albicans* in the urinary bladder of mice as it prevented the adhesion of cell-wall carbohydrates and proteins of *C. albicans* to that of the host [21]. Additionally, Change *et al.* (2012) [22] observed that LL-37 and hBD-3 significantly reduces *C. albicans* attachment to surfaces as it increased the cell-wall β-1,3-exoglucanase Xog1p [22]. They concluded that high levels of β-1,3-exoglucanase Xog1p, which is involved in β-glucan metabolism of the cell-wall, decrease adhesion thus preventing initiation of infection [22]. Another AMP that demonstrated antimicrobial activity against a broad range of Gram-positive, negative, and fungal strains is KABT-AMP [23]. Thankappan *et al.* (2013) [23] observed that application of KABT-AMP led to a survival rate of 31%–32%, in *Escherichia coli*, *S. aureus*, and *C. albicans*, respectively [23]. Additionally, Theberge *et al.* (2013) [24] determined that *C. albicans* growth and transition to its hyphal form was down-regulated as KSL-W analogue was added at two, four, and six days of culture, respectively [24]. Altogether, several synthetic antimicrobial peptides have the potential to prevent *C. albicans* infection as they target the polysaccharides of its cell-wall. However, this is a new research area that needs to be expanded as to what other peptides have therapeutic effects against *C. albicans* biofilm formation and subsequent infection.

#### Antifungals

In a similar arena of treating catheter-related BSIs caused by the commensal fungus *C. albicans*, work has been done to evaluate the effectiveness of antifungals as a preventive solution. Martinez *et al.* (2010) [2] described that the polymer chitosan, which was isolated from crustacean exoskeletons, reduced the cell viability and metabolic activity in *C. albicans* biofilms *in vitro*, and biofilm inhibition *in vivo* [2]. As a hydrophilic polymer, chitosan is created as a result of N-deacetylation of crustacean chitin that is able to penetrate fungal cells by damaging negatively charged cell membranes [2]. Using caspofungin, Lazzell *et al.* (2009) [25] observed a significant reduction of the *C. albicans* in the catheters *in vivo* up to approximately 4 log_10_ [25]. Among the antifungals that were tested, caspofungin was the most effective agent. It inhibits the synthesis of the *Candida* cell wall component, β 1,3-glucan, and the cell walls were observed to be damaged when examined by confocal microscopy [8,14].

A limited number of antimicrobial and antifungal therapies have shown a decrease in BSIs. It was found that even though antimicrobials and antifungals seem to prevent biofilm formation by *C. albicans*, the level of reduction is low and often regrowth occurs and resistance is observed. Thus, several criteria regarding biofilm nature should be considered to reach an effective treatment. Since biofilms are complex and heterogonous structures, it is preferred to apply inhibitory agents that possess a wide spectrum of activity against diverse bacterial and fungal species. To avoid drug resistance, a combination of antimicrobials is suggested if this strategy must be applied [16]. Considering the ineffectiveness of these existing strategies, alternative treatments are sought.

## 4. Potential Approaches

Lack of success in treating catheter-related BSIs with antimicrobials and antifungals has urged the scientific community to propose alternative strategies. These strategies have been reported to control biofilm formation. Currently antimicrobial lock technique (ALT), ethanol application, chelating agents, and biofilm dispersion compounds have been studied. Of these four approaches, ALT and ethanol addition have been receiving most attention, while more work is encouraged to expand our knowledge on chelating agents and biofilm dispersion mechanisms and their role in inhibiting *C. albicans* biofilm formation in catheters (see Table 1).

**Table 1:** Dispersal factors affecting *C. albicans* biofilm.

Factor	Organisms	References
Nutrient starvation	*Shewanella oneidensis* MR-1, *Acinetobacter spp.*, and *Pseudomonas spp.*	Thormann *et al*. 2005; James *et al.* 1995; Barraud *et al*. 2006
Nutrient rich	*Serratia marcescens*	Rice *et al.* 2005
Electron source disruption	*Pseudomonas spp.*	Barraud *et al*. 2006
QS	*Pseudomonas spp.*, *and Candida albicans*	Barraud *et al*. 2006; Rice *et al.* 2005
EDTA	*Pseudomonas aeruginosa*	Banin *et al*. 2006
*Unsaturated fatty acids: cis-2-decanoic* acid (DCA)	*Candida albicans Escherichia coli*, *Klebsiella pneumonia*, *Proteus mirabilis*, *Streptococcus pyogenes*, *Bacillus subtilis*, and *Staphylococcus aureus*	Boon *et al*. 2008; Davies and Marques 2009
*Unsaturated fatty acids:* diffusible signal factor (DSF)	*Candida albicans*, *Pseudomonas aeruginosa*	Boon *et al*. 2008; Wang *et al*. 2004; Davies and Marques 2009
*Unsaturated fatty acids:* farnesol	*Candida albicans*	Boon *et al*. 2008

### Antimicrobial Lock Technique and Ethanol Application

ALT has been used to eliminate the biofilm communities consisting of gram-positive, gram-negative, and fungal cells [26]. Through this technique, a high concentration of an antimicrobial solution is instilled inside the infected catheter to sterilize the device. Toulet *et al.* (2012) [26] applied a concentration of 1000 mg/L of liposomal amphotericin B (L-AMB) that was able to inhibit biofilm activity of a several *Candida* species for up to 48 h after the end of the lock therapy [26]. However, a complete removal of the biofilm was not obtained in this study. Although the technique mentions antimicrobial lock, other solutions such as inhibitory ethanol treatment have also been tested. A promising study, which evaluated ethanol-based and trisodium citrate (TSC) catheter lock solution against several types of microorganisms, concluded that 60% ethanol therapy completely eradicated biofilms formed with *C. albicans* and gram-negative bacilli after 20 min [27]. In contrast, the application of 46.7% TSC only showed a decrease in *C. albicans* biofilm after 24 h [27]. Ethanol lock solution was as successful in biofilm eradication as ethanol was able to denature proteins and cause membrane leaks combined with the instilled flow of the solution.

Despite the high effectiveness of ALT, there are a number of concerns with the approach. Sufficient care is needed, when setting up the antimicrobial lock method, since a leak in the catheter can flush toxic concentrations of the antibiotic agent into the systemic circulation of the patients leading to toxicity and possibly death [27]. Another concern is that flushing high concentrations of strong antimicrobial agents can lead to antimicrobial resistance depending on the mechanism of the applied product [27]. Considering these concerns, ethanol could be the most optimal lock solution, because of its antimicrobial activity against a broad spectrum of microorganisms, its low cost, and absence of resistance [27].

Knowing the inhibitory activity of ethanol, Mukherjee *et al.* (2006) [28] investigated the ethanol-dependent pathway in *Candida* biofilms [28]. They reported that alcohol dehydrogenase (ADH) was down-regulated in *Candida* biofilms when analyzed by proteomics, Western and Northern blotting. Further, they observed, when ADH was down-regulated using disulfiram and 4-methylpyrazole, that a denser *C. albican*s biofilm was formed that strengthened its ability to invade the host tissues. In case of an *adh1* mutant stain, less ethanol but more acetaldehyde was formed compared to the wild-type stains. Additionally, Mukherjee *et al.* (2006) [28] reported that ethanol treatment was effective in reducing the biofilm biomass made by *C. albicans* (*p* < 0.05), but not by *Staphylococcus spp*. (*p* > 0.05) in a rabbit model of catheter biofilm suggesting that ethanol treatment specifically targets *Candida* biofilm formation [28]. As inhibitory effects of ethanol concentrations of 10%, 20%, and 80% were evaluated, similar reduction rates in dry biofilm weight and biomass were seen [28]. This result indicates that ethanol concentrations as low as 10% could be equally effective in inhibiting biofilm formation in *C. albicans*. Even though it has been seen that ethanol treatment can reduce the biofilm activity of *C. albicans*, further research regarding its effectiveness against biofilms of other microorganisms in a polymicrobial model, with different concentrations, and various catheter material types is necessary.

#### Recent Treatments: Chelating Agents and Biofilm Dispersants

Chelating agents may destabilize the biofilm structure, and some of them have shown to have antimicrobial properties against bacteria and fungi. Venkatesh *et al.* (2009) [29] examined the synergistic application of catheter lock solutions of Ethylenediaminetetraacetic acid (EDTA), *N-acetylcysteine* (NAC), talactoferrin (TLF), and ethanol alone or in combination with antibiotics [29]. The study showed that chelating agent in combination with antibiotics was effective against biofilms of *C. albicans* and *Staphylococcus epidermidis* in catheters. These agents are originally known to have inhibitory effects that allowed them to synergistically act as anti-biofilm agents. EDTA inhibits planktonic *Candida* and *Staphylococci*, NAC disrupts EPS formation, and TLF has antimicrobial activity [29]. For all tested 8 mg/mL of EDTA, NCA, TLF, respectively, and 12.5% of ethanol decreased both the mean biofilm mass and thickness in mono species and polymicrobial *C. albicans* biofilms [29]. Among the above listed agents, TLF was the least effective, while ethanol was more successful in reducing *C. albicans* biofilms [29]. The use of chelating agents resulted in substantial change in the biofilm structure suggesting that this treatment has potential for eradication of *C. albicans* biofilms from catheters.

Another treatment strategy aimed at reducing *C. albicans* biofilm is dispersal and subsequent shedding of the fungal cells. A range of factors have shown to induce biofilm dispersal for multiple microorganisms such as harsh physical environments, limited nutrient availability, and quorum-sensing (QS). However, it is not entirely understood whether the detachment occurs due to a controlled biological process or due to sporadic environmental stimuli. It has been suggested that biofilm dispersion can assist in disrupting the *C. albicans* biofilm resulting in planktonic cells that subsequently can be targeted with antifungals and thus removed from the catheter. While this phenomenon needs further research in *C. albicans*, Thormann *et al.* (2005) [30] stated that detachment in biofilms formed by microorganisms, such as *Shewanella oneidensis* MR-1, *Acinetobacter spp.*, and *Pseudomonas spp.* was triggered as a result of nutrient starvation or electron source disruption [30,31,32,33]. The QS disruptor and chelating agent, EDTA was observed to cause cell lysis, loss of cell viability, and increase in cell sensitivity [34]. EDTA not only dispersed the *Pseudomonas aeruginosa* biofilm, it also killed the bacteria [34].

Application of QS inhibitors causing biofilm dispersion needs to be thoroughly examined due to the heterogeneity of biofilms. Currently, few research studies investigated the impact of the QS mechanism on sloughing of *C. albicans* biofilms, but studying the behavior of other microorganisms might provide us with hints to treat or prevent the biofilm formation. Rice *et al.* (2005) [35], indicated that nutrient-rich conditions induced a QS-dependent detachment in biofilm of the opportunistic pathogen, *Serratia marcescens* [35]. Studies characterized a number of QS disruptors, such as *cis-2-decanoic* acid (DCA), diffusible signal factor (DSF), and farnesol that inhibited the germ tube formation (mycelia) by *C. albicans*, respectively, in the order of most effective to least effective [7]. In order to understand the mechanism relating to these unsaturated fatty acids, it is important to note that typically *C. albicans* exist in a yeast form, but the fungus can also form germ tubes (hyphae) that are able to enter bloodstream in humans and cause infections [36]. DCA and DSF have similar structures verified by HPLC and NMR, and DSF is known to be a QS signal for cell communication in a range of bacterial species [7]. DCA significantly decreased the hyphal formation of *C. albicans* by 15% [7].

Although the molecular mechanisms of DCA function needs more investigation, the ability of DCA to target cross-kingdom interactions may provide insights into treating biofilm infections caused by *C. albicans*. Similarly, Davies and Marques (2009) [37] identified that DCA, made by *P. aeruginosa*, could disperse biofilms of *C. albicans* *in vitro* as well as biofilms formed by *Escherichia coli*, *Klebsiella pneumonia*, *Proteus mirabilis*, *Streptococcus pyogenes*, *Bacillus subtilis*, and *Staphylococcus aureus* [37]. It was suggested that application of DCA degraded the EPS of biofilms, which could then be followed by antimicrobial agents to kill the planktonic single cells [37]. Cell-cell communication signals seemed to be one of the promising strategies to target in order to control infectious diseases caused by biofilms. The active detachment of cells from the biofilm mode of growth is helpful in decreasing the BSIs. However, the factors triggering this mode of growth or prevention hereof are not completely understood. After biofilm dispersal, further treatment with antimicrobial agents could effectively eliminate the biofilm presence on indwelling devices, such as catheters, prevent regrowth on the catheter, and thereby improve patient’s lives.

## 5. Conclusions

Intravascular catheters are widely used in medicine, allowing for administration of intravenous fluids, blood, medications, and nutrition. However, their use is associated with a high risk of BSIs caused by colonization of microorganisms in biofilms. One of these organisms, *Candida albicans*, has shown to be one of the leading organisms responsible for these infections by forming biofilms. Polymicrobial biofilms of *Candida* species together with bacteria add to the complexity of the biofilm situation, which complicates the prevention strategies. *C. albicans* is a commensal organism possessing characteristics that allows it to survive in harsh environments and to be flexible in changing from a harmless commensal to an invasive and virulent pathogen. This organism can form highly resistant biofilms in catheters increasing its pathogenicity, which has been difficult to target by solely applying antimicrobials and antifungals. This low success rate in treatment of these infections using antimicrobials is pushing the need for alternative prevention and treatment strategies.

Strategies, such as ALT, chelating agents, ethanol, and QS disruptors appear to be the most promising prevention approaches for catheter-related BSIs. Application of ethanol in ALT has reportedly been the most effective method in preventing BSIs. On a proteomic level, Mukherjee *et al.* (2006) reported that disruption of the protein, *adh1p*, that produces ethanol prevented biofilm formation in *C. albicans* through an ethanol-dependent pathway. Additionally, chelating agents may destabilize the biofilm structure, and some of them have been shown to possess antimicrobial properties against bacteria and fungi. However, caution must be exercised so that application of these agents do not lead to resistance or reduced tolerance. Current efforts lack the ability to present a combined ALT approach with ethanol and a chelating agent that is both effective and harmless to the human body upon administration.

Despite the high effectiveness of ALT, there are a number of concerns with the approach. Sufficient care is needed when setting an antimicrobial lock method, since a leak in the catheter could flush toxic concentrations of the antibiotic agent into systemic circulation of the patient leading to toxicity and possibly death. Another concern is that flushing high concentrations of strong antimicrobial agents could lead to antimicrobial resistance. Considering these concerns, ethanol could be the most optimal lock solution, because of its antimicrobial activity against a broad spectrum of microorganisms, its low cost, and its lack of resistance build-up. 

Despite the high incidence rate, BSIs are preventable and effective strategies are needed to make progress toward the goal of eliminating BSIs. There is a great need to provide an optimal strategy to prevent catheter-related bloodstream infections associated with *C. albicans*. After all, development of a novel strategy that could effectively eliminate biofilm formation on indwelling devices and prevent growth of microorganisms on catheters and thus improve the patient’s quality of life and life-span is necessary.
